# Aberrant XIST beyond the inactivated X chromosome modulates the intracellular dysregulation in Alzheimer’s disease

**DOI:** 10.1002/alz.087394

**Published:** 2025-01-03

**Authors:** Somenath Sen, Debashis Mukhopadhyay

**Affiliations:** ^1^ Homi Bhabha National Institute, Mumbai, Maharashtra India; ^2^ Saha Institute of Nuclear Physics, Kolkata, West Bengal India

## Abstract

**Background:**

Recent advances in understanding the regulatory networks implicated in Alzheimer’s Disease (AD) evinces the involvement of long non‐coding RNAs (lncRNAs) as crucial regulatory players. The present study explores the role played by maternally imprinted lncRNA XIST in regulating the sex‐biased prevalence of AD.

**Method:**

With whole transcriptomic sequencing data from the hippocampal RNA of post‐mortem AD brains from humans and APP/PS1 mice, the altered expression of XIST in AD was studied. The data was further validated in an *in vitro* AD cell model. RNA FISH and subcellular fractionation‐qPCR studies were then performed to trace and quantitate the distribution of XIST in nuclear and cytoplasmic compartments. The nuclear XIST in AD was studied for its altered dynamics with H3K27me3 levels using RNA Immunoprecipitation (RIP)‐qPCR. The fate of the cytosolic XIST was further explored using RIP and miRNA‐lncRNA pull‐down experiments using antibody against RBPMS and biotinylated mimic of miR‐186‐5p.

**Result:**

XIST was found to be significantly upregulated in AD. RNA FISH experiments revealed that XIST from the inactivated X chromosome (Xi) was getting translocated to the cytoplasmic compartment in AD. Upon quantification of the transcript by subcellular fractionation/qPCR, it was found that the heightened XIST expression in AD was due to the ectopic upregulation of the lncRNA in cytoplasm relative to the nucleus. Nuclear XIST on the other hand showed downregulation in AD compared to the control sets. ICC+FISH and RIP studies showed altered dynamics between H3K27me3 levels and XIST in the nucleus in AD. The ectopic XIST was found to be interacting with RBPMS and miR‐186 and knocking down XIST showed opposing effects in the expression levels of RBPMS and miR‐186 in AD. Moreover, RBPMS mRNA was found to be regulated by miR‐186 forming a competitive endogenous RNA (ceRNA) triad modulated by the lncRNA XIST in AD.

**Conclusion:**

This study reports the presence of XIST in cytoplasm in AD for the first time and sheds light on its downstream mechanisms mediated by this aberrant translocation. Studying XIST from the context of AD further allows us to establish the role of this imprinted lncRNA on the gender specific prevalence of AD.

